# Elucidating the influence of supervisors’ roles on implementation climate

**DOI:** 10.1186/s13012-019-0939-6

**Published:** 2019-10-25

**Authors:** Alicia C. Bunger, Sarah A. Birken, Jill A. Hoffman, Hannah MacDowell, Mimi Choy-Brown, Erica Magier

**Affiliations:** 1 College of Social Work, The Ohio State University, 1947 College Road, Columbus, OH 43210 USA; 2 Department of Health Policy and Management, Gillings School of Global Public Health, University of North Carolina at Chapel Hill, 1105C McGavran-Greenberg Hall, Campus Box 7411, Chapel Hill, NC 27599 USA; 3 School of Social Work, Portland State University, 1800 SW 6th Avenue, Suite 600, Portland, OR 97201 USA; 4 Bureau of Maternal, Child and Family Health, Ohio Department of Health, 246 North High Street, Columbus, OH 43215 USA; 5 School of Social Work, University of Minnesota, Peters Hall, 1404 Gortner Ave, Saint Paul, MN 55108 USA

**Keywords:** Supervisors, Implementation, Middle managers, Implementation climate

## Abstract

**Background:**

Supervisors play an essential role in implementation by diffusing and synthesizing information, selling implementation, and translating top management’s project plans to frontline workers. Theory and emerging evidence suggest that through these roles, supervisors shape implementation climate—i.e., the degree to which innovations are expected, supported, and rewarded. However, it is unclear exactly how supervisors carry out each of these roles in ways that contribute to implementation climate—this represents a gap in the understanding of the causal mechanisms that link supervisors’ behavior with implementation climate. This study examined how supervisors’ performance of each of these roles influences three core implementation climate domains (expectations, supports, and rewards).

**Materials and methods:**

A sequenced behavioral health screening, assessment, and referral intervention was implemented within a county-based child welfare agency. We conducted 6 focus groups with supervisors and frontline workers from implementing work units 6 months post-implementation (*n* = 51) and 1 year later (*n* = 40) (12 groups total). Participants were asked about implementation determinants, including supervision and implementation context. We audio-recorded, transcribed, and analyzed focus groups using an open coding process during which the importance of the supervisors’ roles emerged as a major theme. We further analyzed this code using concepts and definitions related to middle managers’ roles and implementation climate.

**Results:**

In this work setting, supervisors (1) diffused information about the intervention proactively, and in response to workers’ questions, (2) synthesized information by tailoring it to workers’ individual needs, (3) translated top managements’ project plans into day-to-day tasks through close monitoring and reminders, and (4) justified implementation. All four of these roles appeared to shape the implementation climate by conveying strong expectations for implementation. Three roles (diffusing, synthesizing, and mediating) influenced climate by supporting workers during implementation. Only one role (diffusing) influenced climate by conveying rewards.

**Conclusions:**

Supervisors shaped implementation climate by carrying out four roles (diffusing, synthesizing, mediating, and selling). Findings suggest that the interaction of these roles convey expectations and support for implementation (two implementation climate domains). Our study advances the causal theory explaining how supervisors’ behavior shapes the implementation climate, which can inform implementation practice.

Contributions to the literature

- Research shows that supervisors and other middle managers play an important role in implementation by shaping the implementation climate (i.e., shared belief that implementation is expected, supported, and rewarded).
- We found that supervisors fulfilled four roles during implementation: they diffused and synthesized information, mediated between strategy and day-to-day tasks, and justified implementation. Together, these roles interacted to convey expectations and support for implementation at the front lines.
- We did not find a robust evidence of how supervisors convey rewards for implementation.
- These findings fill the gaps in knowledge about the specific ways in which supervisors influence implementation.

## Background

Successful implementation of new treatment interventions is dependent on a strong and supportive implementation climate within organizations or the shared perception that an intervention is expected, supported, and rewarded [1–4]. According to Klein, Conn, and Sorra’s Theory of Innovation Implementation, implementation climate is shaped by organizational resources and strategies which are often determined by top-level executive leaders. Supervisors, and other middle managers, bridge organizational hierarchies and are therefore essential for translating executive-level expectations, supports, and rewards for implementation to the front lines. Indeed, empirical evidence suggests that supervisors may have a direct influence on their unit or team’s implementation climate (e.g., [5, 6]). However, the underlying causal mechanisms that link supervisors’ behavior to the implementation climate are unclear.

Drawing from the Theory of Middle Managers’ Roles in Healthcare EBP Implementation, supervisors may influence the implementation climate at the front lines by fulfilling several middle management roles [7]. By sharing and helping their supervisees make sense of information, translating organizational strategies into day-to-day work tasks, and championing implementation, supervisors influence the implementation climate [7–11]. Although recent evidence and theory suggest a link between supervisors and implementation climate, it is unclear how each of these roles conveys expectations, supports, and rewards for implementation (i.e., the causal mechanisms underlying the empirical link between supervisors’ roles and implementation climate) [11]. As a result, there is a limited understanding of specific strategies supervisors might use to create a strong implementation climate, and in turn, implementation and client outcomes.

Set within the context of a large public child welfare agency, this study explored how supervisors’ roles influence implementation climate at the front lines of a high-stress and resource-constrained working environment, where implementation strategies must be feasible and effective. Our study is intended to inform the refinement of causal theories (e.g., Theory of Middle Managers’ Roles in Healthcare EBP Implementation), by identifying practical strategies that supervisors use to promote implementation and exploring how these strategies influence the degree to which the intervention is expected, supported, and rewarded at the front lines of their organization (the three domains of implementation climate). First, we consider the evidence suggesting the importance of a strong implementation climate for supporting proficient use of new interventions and extant theory that explains how supervisors may shape the implementation climate.

## Implementation climate

Implementation climate is an organizational or group-level construct that reflects three domains—the degree to which professionals share the belief that a specific innovation is (1) expected, (2) supported, and (3) rewarded [3]. Implementation climate is a strong predictor of an organization’s consistent and quality use of an innovation [1, 2, 12, 13]. Unlike the organizational climate (which references general perceptions of the work environment), implementation climate strategically focuses on the context for a specific intervention. Staff in the same organization or team may perceive a positive climate for implementing one intervention and a negative climate for implementing another. A strong implementation climate is driven by implementation strategies and policies (set by top managers)—ideally, by providing resources, training, and incentives; helping to “fit” the intervention into existing practice; and removing barriers, organizations theoretically set expectations, provide support, and reward staff for using the new intervention proficiently [2, 3].

Although top-level managers set the implementation strategies and policies, emerging evidence highlights how the implementation climate can vary across teams or units in an organization, which may be attributed to the differences in supervisors’ behaviors and skills. In teams with a stronger implementation climate, supervisors tended to emphasize and spend more time covering evidence-based treatments [5, 14]. Also, more competent, proactive, and transformational leadership approaches by supervisors within teams and units generated stronger and more supportive implementation climates [15–17]. These prior studies indicate that supervisors can positively or negatively influence the implementation climate (given the same organizational supports and policies). However, the specific ways in which supervisors convey that an intervention is expected, supported, and rewarded (i.e., shape the implementation climate) are unclear.

## Supervisors’ roles in implementation

Frontline supervisors serve essential administrative, educational, and supportive functions within health and human service settings as they “direct, coordinate, enhance, and evaluate” frontline professionals’ performance (p. 11) [18]. Generally, high-quality and proactive supervision builds workers’ practice competency, mediates the stress of emotionally burdensome work, and has been linked to a variety of outcomes including satisfaction, retention, and overall performance [19–22]. Specifically, during implementation, supervisors are highly influential as they are one of the most common sources of advice about an intervention and its implementation [23]. When supervisors support and use interventions as intended, frontline professionals are more likely to adopt and adhere to intervention protocols as well [24, 25]. However, prior studies have demonstrated variation and gaps in supervisors’ skills when helping frontline professionals implement a new intervention across a variety of settings [5, 26–28], which may explain variations in implementation climate across teams.

The Theory of Middle Managers’ Role in Healthcare EBP Implementation suggests that variations in the way supervisors (and other middle managers) carry out four critical roles directly shape implementation climate, which in turn influences the implementation success [7, 10]. First, supervisors obtain and *diffuse* information, facts, support, and praise. Second, supervisors *synthesize* that information by interpreting, adapting, and making it relevant to frontline professionals. Third, supervisors *mediate* between top management’s strategy and day-to-day activities by identifying tasks and monitoring performance. Finally, supervisors *sell implementation* by justifying implementation and encouraging intervention use.

**Fig. 1:**
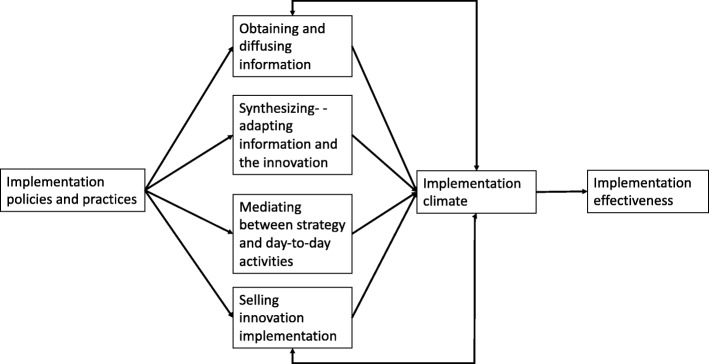
Refined Theory of Middle Managers’ Roles in implementing innovations [11]

Theoretically, by performing these four roles, supervisors can directly influence the implementation climate [11, 29] (Fig. 1). By diffusing and synthesizing information, mediating between top management strategies and everyday practice, and selling implementation, supervisors can directly shape how frontline professionals perceive that an intervention’s use is expected, supported, and rewarded. For instance, supervisors may convey expectations and norms for implementation while diffusing and synthesizing information [30]; supervisors support and reward innovation use through ongoing coaching, feedback, encouragement, and breaking the work down into manageable tasks [31]. Prior research suggests that all four roles are considered important [10] and may build on and reinforce one another to promote implementation success [9]. However, while theory and evidence suggest a link between these roles and implementation climate, we do not know how each role and the underlying mechanisms of action shape how innovation use is expected, supported, and rewarded (the three implementation climate domains). Therefore, this study is intended to address this gap and elucidate how supervisors shape the implementation climate. Specifically, we examined how supervisors fulfill middle managers’ roles (diffusing, synthesizing, mediating, and selling) during the implementation of a new behavioral health intervention and how these roles target the three implementation climate domains (expectations, supports, and rewards) at the front lines.

## Methods

### Study context

This qualitative study was conducted in the context of the Gateway CALL system demonstration project intended to improve access to mental health services for children and youth who enter foster care. The research team was contractually partnered with a county-based public child welfare agency located within an urban midwestern state (USA) that employs about 700 staff and serves about 30,000 children and families with a $200 million (USD) operating budget annually. At the time of the study, the agency was structured hierarchically, with up to 3 to 5 administrative levels separating frontline workers from the top clinical and executive directors. Communication about organizational changes typically flowed in a top-down fashion [32]. Similar to many other public child welfare agencies, this agency experienced substantial stress due to community demands, changing policies, and worker turnover. The intervention conceptualized frontline child welfare workers as “gateways” to specialty mental health services [33, 34] and involved the implementation of a phased service cascade intervention that included the introduction of four components. First, a new behavioral health screening process was implemented within child welfare intake units. Workers in these units investigated child maltreatment allegations and assessed whether family safety risks required intervention. A positive behavioral health screen at intake triggered a behavioral health assessment and referral to treatment by a co-located behavioral health team from a local non-profit organization (the second and third intervention components). The behavioral health assessment team members provided behavioral health consultation, assessment, and linkage support to child welfare agency workers. The fourth component involved case monitoring practices by ongoing child welfare workers to ensure that children received the services recommended by the assessment team. Ongoing workers link families with an array of services, work with caregivers, and assess their progress.

The intervention was rolled out in phases across intake units (and by extension, throughout the behavioral health team, and ongoing child welfare units); the first four intake units began implementing the new screening procedures in February 2015, and the second group of 4 intake units began implementing in October 2015. About 250 frontline staff and supervisors from the agency were involved. The intervention and its implementation have been detailed elsewhere [35, 36]. This study drew from focus group discussions held throughout implementation designed to identify barriers and facilitators to using the intervention components as intended. All study procedures were reviewed and approved by the first author’s Institutional Review Board. For purposes of transparency, we described the methods, results, and interpretations consistent with the Consolidated Criteria for Reporting Qualitative Research [37] (Additional file 1).

### Participants

Study participants included 83 unique child welfare workers (from intake and ongoing units), behavioral health clinicians, and their supervisors in intervention implementing units (about one third of all staff involved in implementation). Most were female (80%), and worked in their position for an average of 5 years (although this ranged from 9 months to 31 years). In terms of their highest degree, half of the participants held bachelors’ and half held master’s degrees. To recruit a criterion sample of participants representing all implementing units and roles [38], the research team collaborated with the agency. Specifically, project leaders at the agency asked the supervisors of the implementing units to identify staff with experience implementing the intervention components and invite them to attend voluntary focus groups. While these methods precluded us from tracking all those recruited, these procedures yielded 51 participants in 2015 and 40 participants in 2016; only 8 individuals participated in both years. The composition of the groups shifted over time due to the expansion to new units (and expanding the number of individuals with exposure to the intervention) and high turnover in the agency (Table 1). Because of our recruitment methods, it is unclear how many individuals were invited but did not participate.

**Table 1:** Focus group participants by role in 2015 and 2016

	July 2015 (6 groups), total	July 2016 (6 groups), total	Participated 2016 only, total	Participated both years, total	Unique participants, total
Behavioral health clinicians	7	4	4	0	11
Intake workers (2 groups)	14	15	11	4	25
Ongoing workers (2 groups)	18	13	11	2	29
Supervisors	12	8	6	2	18
Total	51	40	32	8	83

### Data collection procedures

Twelve focus groups were conducted with 4 to 12 participants in each group; 6 groups were conducted in July 2015 (6 months after the initial implementation; *n* = 51), and 6 were conducted in July 2016 (18 months after the initial implementation; *n* = 40). The groups were homogenous in terms of participants’ unit type (e.g., intake, behavioral health, ongoing) to explore differences and similarities in participants’ perspectives depending on their unique role in the intervention. Also, to encourage participants to share their honest perceptions without fear of potential evaluation or retribution, separate groups were held for those in frontline positions and supervisors. To protect participants’ privacy and keep their conversations confidential, no other individuals besides the participants and research team members were permitted to join the focus group. Lunch was provided as an incentive.

All focus groups were conducted on-site at child welfare or behavioral health agency offices and lasted about 90 min. Informed consent was obtained at the beginning of the focus groups. The discussions were facilitated by research team members including the lead evaluator, project coordinator, and student research assistants, all of whom were female, held or were working towards master’s degrees in social work, and were trained in focus group facilitation. At least three research team members facilitated each group (a facilitator, co-facilitator, or note-taker/observer). The research team members were familiar with the project, the involved agencies, and main project leaders. Likewise, some of the participants were likely somewhat familiar with at least two of the research team members due to prior meetings to discuss implementation and evaluation planning. To build rapport, each research team member introduced themselves briefly at the beginning of the discussion, explained their role on the team, and shared how the team will share general themes from the focus group to evaluate and refine the project’s implementation plans.

To understand how workers’ implementation experiences evolved over time during implementation, both sets of focus groups (in 2015 and in 2016) followed the same semi-structured interview guide focused on implementation (Additional file 2). The guide was developed in collaboration with the agency partners to elicit participants’ views about the new screenings and assessments, changes to their practice, and how the tools were implemented and supported throughout the system. The guide was also pilot tested before the first focus groups with the research team. Discussions were audio-recorded and professionally transcribed. Each facilitator typed up any field notes and also wrote a summary of their impressions after each group.

### Analysis

Transcripts were analyzed using a multi-step process and managed using Atlas.ti 6 and Dedoose. First, transcripts were analyzed using an inductive coding approach consistent with a modified grounded theory approach [39]. An initial codebook was developed based on the interview guide and impressions from the first set of focus groups in 2015. The codebook was refined throughout three coding cycles where two coders independently applied the code book to one transcript, compared their results, resolved coding disagreements with the assistance of a third member of the research team, and adjusted the code book. This final codebook was applied to all other transcripts from 2015 and 2016 by two independent coders who met to discuss the discrepancies. Coders reached at least 80% agreement on all transcripts. At this stage of analysis, we shared a one-page brief with all participants summarizing general focus group themes and invited their feedback [40]. During this member-checking phase, feedback from participants suggested that they felt heard during the focus groups.

Next, one code “Supervision” (*n* = 87 excerpts; about 45 pages of single-spaced text) was further extracted for deductive coding. A second codebook was developed based on concepts, domains, and definitions found in seminal papers on middle managers’ roles [7] and implementation climate [3] (Additional file 3). Finally, we constructed a conceptual matrix to identify and examine excerpts about the relationship between the specific middle management roles (as enacted by supervisors, herein referred to as “roles”) and the conceptual domains of implementation climate [41]. Excerpts for each code were reviewed and discussed by the team for interpretation; quotes were selected to illustrate each major theme (minor edits were made for readability and brevity purposes).

## Results

### What do supervisors do during implementation?

Participants described examples of how supervisors filled all four middle management roles during implementation. Throughout the conversations about supervisors, and regardless of what type of role was described, participants emphasized the importance of daily in-person communication during regular staff meetings, one-on-one supervision, other formal or informal meetings, or via email and phone. Participants reported feeling as though they were “constantly talking about” the intervention and its implementation with their supervisors. Supervisors confirmed that regular discussion was intended to help workers learn how to use the new tools, make sense of new procedures, and monitor their workers’ implementation over time. Through these interpersonal interactions, supervisors disseminated and synthesized information, mediated between strategy and day-to-day tasks, and sold implementation. Illustrative quotes are presented in Table 2.

**Table 2:** Quotes illustrating middle managers’ roles during implementation

Supporting quotes	Notes
Diffusing information	
Interviewee 1: *So maybe our training just … didn’t go over the parts we need every day… like how to implement it and the daily processes maybe.* Interviewee 2: *Or we got the training, and you didn’t have one for months.* Interviewee 1: *So then you ask your supervisor, and so whatever they heard is what you do.* (Intake, July 2016)	Example of diffusing information by relaying information to frontline clinicians
*I asked a supervisor today-I said, Oh, are you coming to the Gateway CALL focus group today?” And s/he said, “What’s Gateway?”* (Ongoing, July 2015)	Example of lack of diffusion of information about an innovation
Synthesize information	
*But you also have to make sure that the worker understands what they’re reading because we have different levels of ability and skill levels within the unit. I mean, I’ve got one [worker] who’s been there for almost eight years, and I’ve got one who’s been there four weeks. …if that four weeker was to get one of these [cases], it’s like, okay we’re gonna have to sit down and we’re gonna have to explain the whole thing. So skill level of the worker and their experience goes into it.* (Supervisors, July 2015)	Example of synthesizing information by tailoring information to individual worker
*I’ve had a case where [in-person] wasn’t an option. So [my supervisor] was like, “Well, if you could catch her on the phone, if you could get those questions and do it that way.”* (Intake, July 2016)	Demonstration of troubleshooting challenging cases and adapting to the local context
Mediating between strategy and day-to-day tasks	
*I guess, if things are really followed-up on, like how they’re supposed to be, so my supervisor’s aware that I [have a Gateway CALL case]. She asks, “Do you have it [the assessment] back? What are the recommendations?” Cuz’ then she puts in her notes. “Okay, what’s the next step? Who’s making the referral, you or CALL?” “CALL.” Okay, next month, “Is your kiddo linked with X, Y, and Z?”* (Ongoing, July, 2015)	Example of mediating by identifying specific activities for implementation
*And I, as a supervisor, it’s hard to keep on top of them to make sure they have some of the key points that were mentioned in the Gateway CALL [assessment], so it just depends on that, and sometimes that takes even more time, because I have to decline the case plan because it didn’t include that information.* (Supervisors, 2015)	Illustration of mediating by monitoring implementation and providing feedback
*Even when we have trouble scheduling. Which I did, several times. Or connecting with a caseworker. He was very, hands on in contacting them and just putting that little extra push, getting them to call me* (Behavioral Health, July 2015)	Example of mediating via engaging in implementation activities
Selling implementation	
*I think that the support is up high, but it’s not trickling down, …If it’s that important to them, then educate your workers, your employees on the importance of this program. So that that support trickles down. We have supervisors tell us, “What is this? We don’t know what this is, or why we’re doing it.”* (Behavioral Health, July 2016)	Example of implementation is not well sold within the organization

#### Diffuse information

Throughout the focus groups, participants reported how their supervisors were the primary source of information during implementation. Supervisors actively communicated with their staff about available supports for implementation (e.g., training opportunities, materials), their expectations for implementing the intervention components, and signaled praise and reinforcement when their staff used the intervention as intended. Supervisors also diffused information when responding to workers’ questions. However, especially during the focus groups with ongoing workers, participants described inconsistent diffusion of information among the supervisors in their units, and some were not even familiar with the project. Poor diffusion generated confusion and frustration, which led frontline workers to seek information from other sources or not at all.

#### Synthesize information

Participants described how supervisors synthesized information—helping their staff use general information that they learned in training about the intervention components and their implementation in routine practice. Frontline child welfare workers and behavioral health clinicians relied on their supervisor to make sense of new practice workflows, make decisions about when and how to implement, and integrate information learned from the new tools into practice. Supervisors described synthesizing information by tailoring it based on workers’ abilities, work style, commitment, and attitudes toward the intervention. Synthesis also occurred when supervisors helped workers adapt the intervention and troubleshoot challenging cases which emerged especially among child welfare workers in the intake units administering the behavioral health screens.

#### Mediating between strategy and day-to-day tasks

Participants described several examples of how supervisors facilitated implementation by mediating between agency strategy (implementing the Gateway CALL project) and day-to-day frontline tasks. Supervisors identified and reminded staff of implementation activities (e.g., bringing the electronic tablet to the caregiver’s home to conduct the behavioral health screen, checking the electronic file for completed screens, and filing the assessment results in the case file once completed). Sometimes, workers noted how their supervisors followed up on their reminders during supervision or case reviews, which helped walk workers through the new workflows. Supervisors noted how these reminders and follow-up activities were linked to their efforts to monitor implementation among their staff. Some supervisors (in the intake units and the behavioral health assessment team) kept separate notes and tracking systems that monitored the progress of each case and those in the ongoing units used formal review/approval processes to provide feedback on performance. Supervisors also actively assisted their staff to use the tools and procedures correctly and on time by engaging in implementation activities. These activities tended to involve assistance with connecting and communicating with other staff and caregivers given the importance of regular coordination between work units and caregivers (who provided the responses to the screens and assessments). Notably, some participants mentioned how their supervisors did not remind them or follow up on project implementation, suggesting that mediating behaviors varied by supervisor.

#### Selling implementation

Themes and examples of how supervisors “sold” (or did not sell) the importance of implementation were infrequent. In only 1 of the 12 focus groups, participants reported that their direct supervisors were “fans” or “advocates” of the project, which conveyed its importance. Rather, discussion suggested that especially within the child welfare agency, workers may not have been sold on the project’s rationale, were encouraged to implement to comply with the funders’ expectations (instead of another reason), and that workers may have implemented without understanding the core problems Gateway CALL was designed to address.

### How do supervisors influence implementation climate?

The matrix analysis-identified excerpts where descriptions of supervisors’ roles overlapped with the three conceptual domains of implementation climate (the shared belief that Gateway CALL was expected, supported, and rewarded). Of these excerpts, many highlighted how supervisors’ activities conveyed expectations or supported Gateway CALL implementation. Few excerpts described how supervisors conveyed rewards.

#### Convey expectations

Participants from all groups discussed how supervisors conveyed expectations, when they diffused and synthesized information, and mediated. By diffusing or synthesizing information about Gateway CALL, supervisors communicated expectations (requirements) for completing the new tools and procedures. Especially in the child welfare units, frontline workers noted how their supervisors told them that the new intervention procedures were mandatory when diffusing information, thus setting expectations for practice. Some supervisors also noted that they tailored information to the individual needs of their workers (synthesizing) and described the anticipated outcomes of the project (facilitating children’s access to care, a selling activity) to communicate the project’s priority and value within the agency. These communicated expectations were further reinforced when supervisors mediated between agency strategy and day-to-day activities at the front lines (via identifying tasks, monitoring implementation, and issuing reminders). In fact, many of the excerpts that described how workers perceived expectations also contained examples of how their supervisor engaged in mediation activities. As intake workers described, supervisors used a variety of mediating activities to reinforce expectations for implementing the screening tools such as monitoring their implementation, and issuing reminders:

Interviewee 1: I mean, my supervisor made it kind of mandatory, so it’s like, “You forgot it the one visit. Like, well, guess you got to go back out.” But that’s about it.

Interviewee 2: Our supervisor wrote up on a white board in her office if we had a custody case, and she wrote, “Gave to CALL.” So, we’d kind of check on each other but also see for ourselves.

Interviewee 3: It’s on the face sheet too. They would write, “You need to complete Gateway CALL.” (Intake, July 2015)

While the child welfare intake workers discussed how their supervisors conveyed clear expectations about screening, ongoing workers (in the child welfare agency) described how unclear communication may have negatively impacted the degree to which workers perceived that the intervention was expected. For instance, workers in the ongoing units noted how some supervisors “do not tell us either way” and there was “little to no directive” in their office about whether they were expected to ensure and monitor whether children received the services recommended by the assessment team (the fourth intervention component). In one of the ongoing groups (July 2015), workers described how the absence of clear communication and strong expectations led them to defer to an external behavioral health providers’ discretion rather than follow through on the assessment team’s recommendation.

#### Provide support

Themes related to perceived support for Gateway CALL most commonly emerged during the 2015 focus groups and were linked to descriptions of three role types, highlighting how a variety of supervisory activities helped workers and clinicians feel supported especially at the beginning of implementation. Within the child welfare ongoing units, workers noted how “constant conversation” with their supervisors about the project generally (diffusing activities), reminders, and assistance with applying the new procedures with specific families (synthesizing activities) helped them to feel supported. Supervisors also supported their staff through their mediating activities. For instance, a behavioral health clinician described how her supervisor supported her implementation efforts by monitoring her use of the new assessment tools and assisting with score interpretation (mediating activities).

So [my supervisor], was really involved in that one, really helped with the interpretation and we had a meeting after that and another call when it come through just to kind of review where we were at. And, so there was a lot of support in that first part. (Behavioral Health, July 2015)

There were also instances where workers’ described how even with clear directions from their supervisor, they still felt unsupported because of limited communication with and help from their supervisors. For example, one intake worker (July 2015) shared how they received directions from their supervisor “to show up to this [training] and I had no idea what was going on… and that [the screens] had to be on time. S/he told me to do these custody cases.” In these situations, workers turned to co-workers for the explanation of the intervention’s purpose and support.

#### Reward implementation

Discussion of rewards for implementation was rare, and workers often felt as though supervisors conveyed expectations for task completion without any positive feedback which may have led to confusion about how the intervention should be implemented. As one CALL clinician summarized, “we still don’t know what we can do because the feedback we get is. “We need the data. We have to do it. You have to do it.” Themes about the role of supervisors for rewarding implementation only emerged during focus group discussions with frontline intake workers in 2015. In both focus groups with intake workers, some workers noted that their supervisors diffused verbal praise (e.g., “good job”) when they successfully used the new screening tools and occasionally bought lunch for workers in the unit to reward implementation (diffusing information and rewards). However, intake workers from other units explained that diffusion of rewards and praise for implementation and other work efforts are rare but incredibly powerful for emotionally bolstering those on the front lines during implementation, and in general:

There is not a lot of thanks in our job. People don’t want to see us. People do not like us. You know? … So, when your supervisor does say, like, good job, it’s just like … I did do something right today even though I have 35 voicemails from people screaming at me. You know? … Just having that sometimes is enough to be like, oh, okay. Well, all hope isn’t lost… (Intake, July 2015)

## Discussion

Supervisors can directly influence the implementation climate or the shared sense that an intervention is expected, supported, and rewarded. Yet, we have limited understanding of exactly how supervisors shape the implementation climate. This study drew on the theory of middle managers’ roles in healthcare EBP implementation to examine the relationship between four supervisory roles (diffusing and synthesizing information, mediating, and selling implementation) and implementation climate. Although these roles have been highlighted in other studies, this is the first to examine how they influence the three implementation climate domains (expectations, support, and rewards). Moreover, we observed how supervisors shape the implementation climate in a high-stress and resource-constrained work environment, which illustrates the feasibility and acceptability of carrying out these roles. Thus, our results advance causal theory about how supervisors (and other middle managers) shape implementation climate and implementation practice. Our findings suggest that these roles interact and may especially reinforce expectations and support for implementation. We discuss these findings and their implications for advancing theory, and development of implementation strategies that target or engage individuals in these key roles.

### Supervisors shape the climate during implementation

Consistent with theory and evidence from studies in other organizational settings [9, 29], supervisors in this study filled four roles. Supervisors engaged in mediating activities that translated top management implementation plans to frontline professionals and diffused and synthesized information. Supervisors also “sold” implementation, although they engaged in this role less than other roles perhaps because of competing demands (e.g., “initiative fatigue”) [42] or they felt that the intervention detracts from their focus on child safety (a common issue when implementing behavioral health-focused interventions in child welfare settings) [43, 44]. Nonetheless, these results provide support for the middle managers’ roles specified by theory and the application of this theory to supervisors’ behaviors within child welfare contexts. Our results also confirm that frontline supervisors are appropriate and indeed significant targets for implementation strategies.

Importantly, this study offers preliminary evidence of how supervisors directly influence the implementation climate, as posited by theory. All four of these roles appeared to convey expectations for implementation in this organization, which is one of the implementation climate domains. Carrying out diffusing, synthesizing, and mediating roles also seemed to convey support, particularly during the early stages of implementation. However, there was only limited evidence of how these roles convey rewards for implementation. These findings lead us to several insights that have implications for implementation research and practice.

First, the data suggest that these roles may interact with one another in potentially powerful ways to shape the implementation climate. In fact, these roles seemed to reinforce one another, whereby synthesizing information, mediating (e.g., reminding a worker to conduct the new screening), and selling the importance of the intervention for improving children’s outcomes emphasized basic expectations and information about new mandates that supervisors diffused to their staff. In a similar fashion, during early implementation phases, diffusing information, synthesizing efforts to help workers understand and apply the new intervention procedures, and mediating activities that held workers accountable also helped frontline professionals feel supported, a second implementation climate domain. The interactions among these roles (and how supervisors in this study often carried out more than one role in a given example) highlight the potentially reinforcing effects and raise conceptual questions about whether these four roles are operationally distinct. However, they also warn of the potential for “mixed messaging” if supervisors’ roles are not carried out consistently. For instance, supervisors might diffuse information about an intervention and implementation expectations. Yet, without efforts to monitor how well workers are implementing the intervention (mediating), or help working through difficult cases (synthesizing), workers could feel unsure about what is expected of them or whether there is sufficient support or justification for making these changes to their practice. Consistent execution (across roles and over time) might be especially important in settings with high staff turnover, and thus intensive staff training and support needs. For instance, the median length of employment for a frontline intake worker at this organization during the study period was only 8 months, and supervisors were constantly supporting newly hired frontline staff. Diffusing, synthesizing, mediating, and “selling” efforts that converge around the same message may provide clear and constant support that help newer workers learn their jobs and implement interventions.

Second, our findings may also reflect complex interactions among supervisors’ roles, implementation climate, and general organizational climate, consistent with the evidence from other studies on middle managers [11]. For example, participants from both the child welfare agency and behavioral health team were very attuned to how supervisors conveyed organizational expectations of them (half of the coded climate excerpts referenced expectations), while discussions about how supervisors provided support or rewards were very limited. In fact, supervisors in this study may have carried out their roles with an emphasis on expectations because of the general organizational and child welfare system culture and climate. Child welfare settings are driven by federally mandated benchmarks and time frames for practice [44], which often lead to supervision characterized as “detached and empty” [27] and emphasizing administrative oversight and compliance over education and support [45, 46]. This may explain why there were few examples of how supervisors justified the rationale for the intervention or rewarded a job well done, although future studies examining the nuanced relationship between organizational context, roles, and implementation climate are needed.

Based on the conceptual definition of implementation climate, expectations, support, and rewards are all necessary for generating a strong and shared sense that the organization supports the implementation of an intervention [3]. In settings with compliance-driven cultures that emphasize expectations (especially given the emotionally taxing nature of child welfare practice), supervisors may need to focus more on supporting and rewarding staff during implementation. This may involve explaining the value of the intervention for improving children’s outcomes (a selling activity) and by issuing praise (a diffusing activity). The R^3^ model, for example, trains supervisors on positive reinforcement and praise techniques to improve interactions with frontline workers and between workers and caregivers to promote implementation of new parenting interventions [31]. Support and rewards for implementation might also be conveyed by other organizational stakeholders. Executive leaders can help improve the implementation climate as well by engaging and cultivating leadership among frontline supervisors and aligning how expectations, supports, and rewards for implementation are conveyed [47]. Executive leaders might also need to address general organizational culture and climate issues—approaches such as the Availability, Responsiveness, and Continuity (ARC) model are effective for improving the organizational culture, climate, and implementation [48, 49].

### Limitations

Our findings should be interpreted in light of a few limitations. First, this study relies on self-report, and we did not confirm how supervisors performed each role, their proficiency, or the influence on the implementation climate. Second, while our focus group guide asked about supervision and the general organizational context, we did not specifically ask how supervisors carried out these four roles, or about the implementation climate, so we may not have captured all of the information participants could have offered. Third, our findings about specific implementation roles and activities may have limited generalizability to other health and human service organizations. However, we drew our in-depth data from multiple work units, functions, and points in time and interpreted our results in light of rich contextual information. Our qualitative results provide examples of supervisors’ behaviors in a single context. In the future, these reported behaviors could be triangulated with external observations to test the interrelationships between roles and climate. Ideally, we would examine the causal effects of these different roles on implementation climate by conducting a randomized controlled trial where workers’ exposure to each role was manipulated. However, this type of design and deliberately withholding potentially necessary supervisory roles within a real-world service delivery organization is not feasible. Therefore, other non-experimental approaches are likely to be necessary. This trajectory of research could specify the causal mechanisms that explain how supervisors and other middle managers create a strong and positive implementation climate and directly inform how supervisors support implementation within their teams.

## Conclusion

Supervisors have direct influence over the implementation climate at the front lines and by extension implementation and client outcomes. This study provided preliminary evidence to elucidate how supervisors shape the degree to which an intervention was expected, supported, and rewarded (i.e., the implementation climate). Specifically, we show how supervisors fill four important roles during implementation: they diffuse and synthesize information, sell implementation, and mediate between organizational strategy and day-to-day tasks. Our findings suggest that these roles may interact with one another in potentially powerful ways to shape the implementation climate. They may also reflect complex interactions among supervisors’ roles, implementation climate, and general organizational climate, consistent with the evidence from other studies on middle managers [11]. These results advance the Theory of Middle Managers in Healthcare EBP Implementation and inform how supervisors might facilitate strong conditions for implementation within their teams.

## Supplementary information

**Additional file 1.** Consolidated Criteria for Reporting Qualitative Research (COREQ) Checklist.

**Additional file 2.** Focus group guide.

**Additional file 3.** Codebook

## Data Availability

The data gathered and used for this analysis is not publically available because of the inclusion of identifying information of the participants and potentially sensitive case information.

## Abbreviations

EBP: Evidence-based practice
CALL: Consultation, Assessment, Liaison, and Linkage
