# Supplementary material for: Elucidating the influence of supervisors’ roles on implementation climate
Source: Implement Sci. 2019 Oct 25;14:93. doi: 10.1186/s13012-019-0939-6 (PMC6815002; doi:10.1186/s13012-019-0939-6)
Supplement: Supplementary file 1 — Additional file 1. Consolidated Criteria for Reporting Qualitative Research (COREQ) Checklist. [file 13012_2019_939_MOESM1_ESM.docx]

**Supplemental File 1**

Consolidated Criteria for Reporting Qualitative Research (COREQ) Checklist

| **Criteria** | **Evidence** | **Page Number** |
| --- | --- | --- |
| **Domain 1: Research Team and Reflexivity** | | |
| *Personal Characteristics* | | |
| 1. Interviewer/facilitator  Which author/s conducted the interview or focus group? | Alicia Bunger, Jill Hoffman, Hannah MacDowell, Mimi Choy-Brown | 22 (Authors Contribution) |
| 2. Credentials  What were the researcher’s credentials? E.g. PhD, MD | All held, or were working toward earning masters’ degrees in social work at the time of the study | 10 (Data collection procedures) |
| 3. Occupation  What was their occupation at the time of the study? | Assistant Professor/Lead evaluator,  Project coordinator  Research assistants | 10 (Data collection procedures) |
| 4. Gender  Was the researcher male or female? | Female | 10 (Data collection procedures) |
| 5. Experience and training  What experience or training did the researcher have? | Trained in focus group facilitation | 10 (Data collection procedures) |
| *Relationship with Participants* |  |  |
| 6. Relationship established  Was a relationship established prior to study commencement? | Research team contracted by the agency | 8 (Study context) |
| 7. Participant knowledge of the interviewer  What did the participants know about the researcher? e.g. personal goals, reasons for doing the research | Some early contact during implementation and evaluation planning | 10 (Data collection procedures) |
| 8. Interviewer characteristics  What characteristics were reported about the interviewer/facilitator? e.g. Bias, assumptions, reasons and interests in the research topic | Name, role on project, how they’ll use the information to adjust implementation | 10 (Data collection procedures) |
| **Domain 2: Study Design** | | |
| *Theoretical Framework* | | |
| 9. Methodological orientation and Theory  What methodological orientation was stated to underpin the study? e.g. grounded theory, discourse analysis, ethnography, phenomenology, content analysis Participant selection | Modified Grounded theory – informed by The Theory of Middle Managers Role in Healthcare EBP Implementation, and conceptual definition of implementation climate | 10-11 (Analysis) |
| *Participant Selection* | | |
| 10. Sampling  How were participants selected? e.g. purposive, convenience, consecutive, snowball | Purposive sampling in collaboration with agency | 9 (Participants) |
| 11. Method of approach  How were participants approached? e.g. face-to-face, telephone, mail, email | Supervisors identified and invited individuals to participate | 9 (Participants) |
| 12. Sample size  How many participants were in the study? | 83 child welfare workers, behavioral health clinicians, and supervisors | 9 (Participants) |
| 13. Non-participation  How many people refused to participate or dropped out? Reasons? | Procedures did not allow us to track those recruited but did not participate; only 8 participated in both groups due to expansion to new units and turnover. | 9 (Participants) |
| *Setting* | | |
| 14. Setting of data collection  Where was the data collected? e.g. home, clinic, workplace | On-site at child welfare or behavioral health agency offices | 10 (Data collection procedures) |
| 15. Presence of non-participants  Was anyone else present besides the participants and researchers? | No | 10 (Data collection procedures) |
| 16. Description of sample  What are the important characteristics of the sample? e.g. demographic data, date | 80% female, 50% held BA as highest degree, average of 5 years in position (ranging from nine months to 31 years) | 9 (Participants) |
| *Data Collection* | | |
| 17. Interview guide  Were questions, prompts, guides provided by the authors? Was it pilot tested? | Semi-structured interview guides were used in all focus groups; the guide was pilot tested | 10-11 (Data collection procedures); Supplemental File 2. |
| 18. Repeat interviews  Were repeat interviews carried out? If yes, how many? | Yes – 8 participants participated in focus groups in 2015 and 2016 | 9 (Participants) |
| 19. Audio/visual recording  Did the research use audio or visual recording to collect the data? | Audio recorded | 11 (Data collection procedures) |
| 20. Field notes  Were field notes made during and/or after the interview or focus group? | Field notes, and facilitator observation summaries | 11 (Data collection procedures) |
| 21. Duration  What was the duration of the interviews or focus group? | 90 minutes | 10 (Data collection procedures) |
| 22. Data saturation  Was data saturation discussed? | N/A. Participants were recruited purposively to represent units engaged in implementation (thus theme saturation was not a goal of our sampling approach). | N/A |
| 23. Transcripts returned  Were transcripts returned to participants for comment and/or correction? | Transcripts were not returned, but a one page summary was shared with participants for comment | 11 (Analysis) |
| **Domain 3: Analysis and Findings** | | |
| *Data analysis* | | |
| 24. Number of data coders  How many data coders coded the data? | Two coders per transcript, third coder resolved coding disagreements | 11 (Analysis) |
| 25. Description of the coding tree  Did authors provide a description of the coding tree? | See codebook for supervisory roles and implementation climate | Supplemental File 3 |
| 26. Derivation of themes  Were themes identified in advance or derived from the data? | Both – first round of codes were derived from the data; second round of codes were identified a priori based on theory | 11 (Analysis) |
| 27. Software  What software, if applicable, was used to manage the data? | Atlas.ti 6; Dedoose | 11 (Analysis) |
| 28. Participant checking  Did participants provide feedback on the findings? | One page summary shared | 11 (Analysis) |
| *Reporting* | | |
| 29. Quotations presented  Were participant quotations presented to illustrate the themes / findings? Was each quotation identified? e.g. participant number | Yes | 12-17 (Results)  30 (Table 2) |
| 30. Data and findings consistent  Was there consistency between the data presented and the findings? | Yes | 12-17 (Results)  17-22 (Discussion)  31 (Table 3) |
| 31. Clarity of major themes  Were major themes clearly presented in the findings? | Each major theme discussed under corresponding subheading | 12-17 (Results) |
| 32. Clarity of minor themes  Is there a description of diverse cases or discussion of minor themes? | Where applicable | 12-17 (Results) |

**Reference**

Tong, A., Sainsbury, P., & Craig, J. (2007). Consolidated criteria for reporting qualitative research (COREQ): a 32-item checklist for interviews and focus groups. *International Journal for Quality in Health Care*, *19*(6), 349–357. https://doi.org/10.1093/intqhc/mzm042
