# Supplementary material for: Elucidating the influence of supervisors’ roles on implementation climate
Source: Implement Sci. 2019 Oct 25;14:93. doi: 10.1186/s13012-019-0939-6 (PMC6815002; doi:10.1186/s13012-019-0939-6)
Supplement: Supplementary file 2 — Additional file 2. Focus group guide. [file 13012_2019_939_MOESM2_ESM.pdf]

Supplemental File 2
Gateway CALL Phase II – Years 3 & 4
Focus Group Guide

(with Alicia's prompts and probes)

Pre Conversation

- Welcome! Thank you for volunteering to participate in this focus group.
- The purpose of our conversation this morning is to learn more Gateway CALL.
- Before we introduce ourselves we have a bit of business to address (ground rules) …
- Introductions – around the room
- Any questions before we begin?

Focus Group Conversation

1. Let's begin by talking about the screening and assessment **tools** for Gateway CALL. What do you think of them?
    a. Are they accurate? Easy to use?
    b. Useful? What would make the process more useful?
    c. Have you had trouble using the tools? What kind?
    d. How has this impacted your work-load?
    e. What do you like about the questions/answers? Dislike?

2. What have you had to change about your **practice or routines** to use the new screening and assessment tools?
    a. Have you had to change up your routines? How so?
    b. Have you seen others change their routines to use the new tools successfully?

3. Think back to when you began using these new **tools**. Were there any supports or structures put in place that influenced how you used the new tools? By structures, I mean policies, rules, procedures, resources, or infrastructure.
    a. What has been the most helpful to you for using the tools?
    b. Have these supports been constant or have they changed over time? How so? What has that meant for your practice?

4. How have your **colleagues** influenced how you use the tools? Can you provide an example?
    a. Outside FCCS – with other agencies?
    b. Inside FCCS – with supervisors? Colleagues? Leadership?
    c. What relationships have been most helpful to you as you implement these tools?

5. Have you or your unit made any **adaptations or changes** to the tools or process?
    a. Is everyone using the process similarly within your team? Across units?
        i. How have the procedures changed? What was the rationale for the adjustment?

6. How have the screening/assessment tools impacted your **day to day work**?
    a. How have caregivers reacted to the tools?
    b. How has it impacted your relationship with the CALL team/FCCS workers?
    c. How has it impacted your referrals?
    d. Has it impacted the number of kids who are connected with services? Linkage time?

7. Let's switch gears a bit and talk about **training**. Do you feel that the training on the tools provided you with all of the information necessary to implement the screening/assessment tools?
    a. Is any follow up needed?
    b. What about the training worked well? What could have been different?

8. What **advice** do you have for other agencies who are interested in replicating GWC?
    a. What do you think is most critical for implementing a program like this effectively? What could be done differently?
    b. If you could go back in time, what advice would you give to yourself about Gateway CALL?

9. Summary major points – we talked about the tools and process, what's changed for you, how it's impacted your work, training, and advice you'd give others. **Have we missed anything**?
