# Supplementary material for: Elucidating the influence of supervisors’ roles on implementation climate
Source: Implement Sci. 2019 Oct 25;14:93. doi: 10.1186/s13012-019-0939-6 (PMC6815002; doi:10.1186/s13012-019-0939-6)
Supplement: Supplementary file 3 — Additional file 3. Codebook [file 13012_2019_939_MOESM3_ESM.docx]

**Supplemental File 3: Codebook**

NOTES:

1. Conceptual framework is the theory of middle managers’ role in innovation implementation [1,2]:
2. Make sure to code question as well as response when response does not reflect content of question.
3. Code both affirmative and negative responses.
4. Code both roles and influence of roles on implementation climate

| **Construct/Role** | **Definition/Activities (for roles)*** | **Examples and Rules** |
| --- | --- | --- |
| Implementation climate | “…a shared perception among targeted organizational members that implementation is a major organizational priority – promoted, supported, and rewarded by the organization.” (Helfrich et al., 2007, p.281)[3]  The degree to which an innovation is rewarded, supported, and expected [4,5]. | Used climate if there is an indication that workers share the perception that GWC is expected, supported, rewarded, as a result of the supervisors’ action. |
| Diffusing information | Relay information regarding innovation implementation to employees | Websites  Print/electronic media  Training  Communicating information (e.g., in-person, meetings, email)  Education  Poor information diffusion or lack of diffusion |
|  | Stay attuned to top managers’ and frontline employees’ moods and needs |  |
|  | Provide frontline employees with the information necessary to implement innovations |  |
|  | Provide top managers with feedback regarding innovation implementation status |  |
|  | Field employees’ questions regarding innovation implementation |  |
|  | Inform employees of an innovation that is expected to be implemented |  |
|  | Disseminate information regarding material support for innovation implementation |  |
|  | Disseminate information regarding emotional support for innovation implementation |  |
|  | Disseminate rewards for innovation implementation |  |
|  | Obtaining information |  |
|  | Communicating information to external stakeholders |  |
| Synthesizing information | Make general information about innovation implementation relevant to unique organizations and employees | Workflow integration  Providing examples  Tailoring information to worker skills/needs  Trouble-shooting challenging cases  Limited use of synthesizing information |
|  | Monitor employees’ responses to the information and reinterpret the information in a way that the employee may find more relevant |  |
|  | Use daily conversations to help frontline employees understand key information regarding innovation implementation |  |
|  | Interpret facts about innovation implementation may convey to employees the relevance of the innovation to the specific roles that they are expected to fulfill |  |
|  | Explain to employees the specific ways in which someone in their role would be supported and rewarded for innovation implementation |  |
|  | Adapting innovation to local context |  |
| Mediating between strategy and day-to-day activities | Give employees the tools necessary to implement innovations | Provide data  Funding  Resources  Assuring right equipment / tools  Support  Workflow integration  Reminders / follow-up  Monitoring progress  Helping workers connect/communicate  Not using mediating activities |
|  | Translate information into concrete tasks that must be carried out to effectively implement innovations |  |
|  | Provide employees with practical feedback on their innovation implementation-related performance |  |
|  | Form “strategic communities” that promote the implementation of new technologies |  |
|  | Identify specific activities in which employees are expected to engage to promote an organization’s strategy of innovation implementation |  |
|  | Measuring employees’ innovation implementation-related performance |  |
|  | Engaging in innovation implementation-related activities |  |
| Selling innovation implementation | Justify innovation implementation  Encourage employees to consistently and effectively use innovations | Generating “buy-in” from employees  Providing rewards  Reinforcement  Lack of enthusiasm  Describing benefits |
|  | Convince employees that innovation implementation is worthy of their attention |  |
|  | Set innovation implementation-related norms |  |
|  | Maintain a positive attitude regarding innovation implementation |  |
|  | Help employees to appreciate the rationale underlying organizational changes |  |

**References**:

1. Birken SA, DiMartino LD, Kirk MA, Lee SYD, McClelland M, Albert NM. Elaborating on theory with middle managers’ experience implementing healthcare innovations in practice. Implement Sci [Internet]. 2016;11:1–5. Available from: http://dx.doi.org/10.1186/s13012-015-0362-6

2. Birken S a, Lee S-YD, Weiner BJ. Uncovering middle managers’ role in healthcare innovation implementation. Implement Sci [Internet]. 2012;7:28. Available from: http://www.pubmedcentral.nih.gov/articlerender.fcgi?artid=3372435&tool=pmcentrez&rendertype=abstract

3. Helfrich CD, Weiner BJ, McKinney MM, Minasian L. Determinants of Implementation Effectiveness. Med Care Res Rev. 2007;64:279–303.

4. Weiner BJ, Belden CM, Bergmire DM, Johnston M. The meaning and measurement of implementation climate. Implement Sci [Internet]. 2011 [cited 2011 Nov 17];6:78. Available from: http://www.implementationscience.com/content/6/1/78

5. Jacobs SR, Weiner BJ, Bunger AC. Context matters: measuring implementation climate among individuals and groups. Implement Sci [Internet]. 2014 [cited 2014 Apr 28];9:46. Available from: http://www.implementationscience.com/content/9/1/46
